# Factors affecting the decision to hospitalise children admitted to the emergency department due to non-fatal suicide attempts by pills

**DOI:** 10.12669/pjms.323.9765

**Published:** 2016

**Authors:** Gamze Gokalp, Murat Anil, Alkan Bal, Yuksel Bicilioglu, Fulya Kamit Can, Ayse Berna Anil

**Affiliations:** 1 Gamze Gokalp, MD. Pediatric Emergency Department, Izmir Tepecik Teaching and Research Hospital, Izmir, Turkey; 2 Murat Anil, MD. Associate Professor, Pediatric Emergency Department, Izmir Tepecik Teaching and Research Hospital, Izmir, Turkey; 3 Alkan Bal, MD. Pediatric Emergency Department, Izmir Tepecik Teaching and Research Hospital, Izmir, Turkey; 4 Yuksel Bicilioglu, MD. Pediatric Emergency Department, Izmir Tepecik Teaching and Research Hospital, Izmir, Turkey; 5 Fulya Kamit Can, MD. Pediatric Intensive Care Clinic, Izmir Tepecik Teaching and Research Hospital, Izmir, Turkey; 6 Ayse Berna Anil, MD. Associate Professor, Pediatric Intensive Care Unit, Pediatric Intensive Care Clinic of Izmir Katip Celebi University, Izmir Tepecik Teaching and Research Hospital, Izmir, Turkey

**Keywords:** Hospitalisation, Paediatric, Emergency, Self, Poisoning

## Abstract

**Objective:**

Suicide attempts (SAs) in the paediatric age group represent an important cause of morbidity and mortality. Our aim was to examine the factors affecting the decision to hospitalize children with a diagnosis of non-fatal SA by pills.

**Methods:**

Children <18 years of age admitted with SA by pills during 2014 were evaluated retrospectively. Patients were divided into two groups: Group-I comprised hospitalised patients and Group-II included those who were discharged from the PED. These two groups were compared in terms of clinical and demographic characteristics recorded upon PED admission.

**Results:**

A total of 196 patients were included in the study. The number of pills taken for self-poisoning in Group-I (median: 20 pills) was higher than that in Group-II (median: 12 pills) (p < 0.001), and the rate of pathological findings during the first paediatric psychiatric consultation was higher in Group-I (91.1%) than in the Group-II (54.8%) (p < 0.001).

**Conclusion:**

Factors affecting the disposition decision in cases of children who performed non-fatal SA via pills included the amount of medication taken for the suicide attempt and the presence of psychiatric disorders, as determined by a paediatric psychiatrist during the acute phase.

## INTRODUCTION

Suicide attempts (SAs) represent one of the most important causes of mortality among children and adolescents.^1^ In the United States, suicide has been identified as the third most important cause of death for those between the ages of 10 and 24 years. According to these data, approximately 4300 deaths per year were due to SA. Non-fatal SAs are much more common, comprising about 5% of emergency department visits.^2^

A variety of means may be involved in SA. The most common means are firearms, stabbing, hanging, drowning, motor vehicle injuries, jumping from a high place, and poison ingestion.^3^–^5^ Approximately, 50% of SA cases are hospitalised.^2^ Most studies of SA have focused on psychiatric causes.^2^ Nonetheless, one of the most practical issues from the perspective of the paediatric emergency physician is the decision regarding whether a patient being treated for non-fatal SA should be hospitalised.

Our objective was to determine which demographic, clinic, and laboratory risk factors upon admission to a paediatric emergency department (PED) were influential in the decision to hospitalise children being treated for non-fatal self-poisoning by pills. We chose to focus on self-poisoning by pill ingestion, because this is one of the most common means of SA in our patient.

## METHODS

A retrospective analysis of the records of paediatric patients admitted to the PED of Tepecik Teaching and Research Hospital, Izmir, Turkey, from 1st January to 31st December 2014 was performed. The local ethical committee granted permissions. The PED of Tepecik Teaching and Research Hospital, one of the most crowded PEDs in Turkey, is a paediatric emergency sub-specialty training clinic. The PED has a 14-bed observation unit to monitor and treat critical patients for up to 24 hour, after which the patients can be admitted to the hospital if necessary. The standard interventions (gastric lavage, activated charcoal treatments, and specific antidotes as appropriate) are performed in all patients admitted to the emergency department where they are observed. Consultations with the National Poison Control Centre are routine. A paediatric psychiatrist performs psychiatric consultations with the patients in the PED or the paediatric ward.

Criteria for inclusion in our study were children <18 years of age who came to the PED following self-poisoning by pills. Self-poisoning was defined as intentional ingestion of a toxic substance or medication in excess of the prescribed dosage. Our hospital data system has a standard poisoning and suicide medical record page that must be filled out by the physician who provides medical care. This medical record page includes thorough demographic and medical data. For the present study, we reviewed the suicide-specific medical records in the hospital’s electronic data system to collect data on the patient’s age, gender, and medical history, including the amount of drugs (in this study, the number of pills) taken for self-poisoning, physical examination findings, results of all laboratory tests, results of the first psychiatric evaluation, disposition type, and final outcomes. Patients were divided into two groups according to the disposition type: Group-I included patients who were hospitalised and Group-II included patients who were discharged from the PED. These two groups were compared in terms of clinical and demographic characteristics. Patients who attempted suicide by other means (stabbing, firearms, intentional traffic accidents, intentional choking, jumping, or hanging) were excluded.

Categorical data are expressed as number (n) and percentage (%) and were analysed using chi-square or Fischer’s exact tests, as appropriate. Continuous data are presented as mean±standard deviation (SD) and minimum–maximum values, or as median and interquartile range (25–75^th^ percentile), according to the parametric condition. Student’s t-tests or Mann–Whitney U-tests were used for the analyses of continuous variables. A p-value < 0.05 was considered statistically significant. All statistical analyses were performed using IBM SPSS Statistics 20.0 software (IBM, Armonk, NY, USA).

## RESULTS

During the study period, 161,928 children were admitted to the PED of Tepecik Teaching and Research Hospital, Izmir, Turkey. Of these, 196 patients (mean age: 14.0±1.7, range: 6–17 years; 167 female, 29 male) were included in the study. According to their medical histories, 21 (10.7%) children had one or more previous SAs. Antidepressant drugs (52 patients, 26.5%) were the most commonly used drugs (Table-I), and 97 (49.5%) patients engaged in multi-drug self-poisoning. On admission, 10 patients (5.1%) exhibited abnormal physical examination findings, 44 patients (22.4%) had abnormal laboratory results (22 serum electrolyte abnormality, 27 liver function test abnormality, and four renal function test abnormality), and five patients (2.6%) had abnormal electrocardiogram (ECG) findings (4 sinus tachycardia and one ventricular tachycardia). Low Glasgow Coma Scale (GCS) scores were identified in 6 patients (3.2%) on admission (4 patients had scores of 14; 2 had scores of 13). Paediatric psychiatric consultations were performed for all patients in the PED or paediatrics ward. The first psychiatric consultations identified pathological conditions in 152 (77.6%) patients (Table-II). After observation in the PED, 123 (62.8%) patients were hospitalised (7 patients in the paediatric intensive care unit; no patient required mechanical ventilation). There was no patient who was hospitalized at pediatric psychiatry unit. The median length of stay was 2 days (25–75^th^ percentile: 2–4 days; range: 1–10 days). No patient died. All patients were discharged from the hospital neurologically intact.

**Table-I:** Drugs used in self-poisoning and hospitalization rates in each drug type.

*Type of drug*	*Number of patient (% of total patient)*	*Number of hospitalized patient (% of drug type)*
Antidepressant	52 (26.5)	28 (53.8)
Paracetamol	45 (23)	33 (73.3)
Acetylsalicylic acid	16 (8.2)	11 (68.8)
Antigripal drug	47 (24)	32 (68.1)
Nonsteroidal anti-inflammatory drug	45 (23)	33 (73.3)
Cardiovascular drug	22 (11.2)	17 (77.3)
Anti-diabetic	7 (3.6)	6 (85.7)
Antibiotic	29 (14.8)	19 (62.1)
Anti-histaminic	15 (7.7)	10 (66.7)
Ferrous drugs	8 (4.1)	5 (62.5)
H2 receptor blocker	24 (12.2)	18 (75)
Antiepileptic	15 (7.7)	8 (53.3)
Colchicine	1 (0.5)	1 (100)
Others	15 (7.7)	10 (66.7)

**Table-II:** The results of pediatric psychiatric consultation.

*Result of pediatric psychiatric consultation*	*n (%)*
Major depression	112 (57.1)
Anxiety disorder	22 (11.2)
Pervasive developmental disorder	3 (1.5)
Pathological sadness	4 (2)
Adjustment disorder	7 (3,6)
Mood disorders	4 (2)
No clear pathology	44 (22.4)

We compared the hospitalised patients (Group-I) and discharged patients (Group-II) according to demographic, clinical, and laboratory findings upon admission to the PED. There were no statistical differences between the groups in terms of age, gender, presence of multi-drug poisoning, presence of self-harm scarring, rate of first SA, GCS on admission, pathological findings on physical examination on admission, ECG abnormality on admission, or abnormal laboratory test results on admission (all *p* > 0.05). The number of pills taken for self-poisoning in Group-I was much higher than that in Group-II (median number of tablets: 20 vs. 12; *p* < 0.001). The rate of pathological findings during the first paediatric psychiatry consultation was significantly different between Group-I (112 patients, 91.1%) and Group-II (40 patients, 54.8%) (*p* < 0.001) (Table-III). Furthermore, we found no significant difference in the rate of hospitalisation based on the type of drug used in self-poisoning (*p* > 0.05). The highest hospitalisation rates were seen in children who ingested anti-diabetic medications (17 of 22 patients, 77.3%) and in children with self-poisoning via colchicine (1 patient, 100%) (Table-I).

**Table-III:** The differences between the cases with or without hospitalization according to demographic and clinical findings.

*Parameters*	*Total n: 196*	*Group-I n: 123*	*Group-II n: 73*	*p*
Age, mean±SD (minimum – maximum)	14.0±1.7 (6-17)	14.1±1.6 (9-17)	13.8±1.9 (6-17)	0,235
*Gender, n (%)*				
F	167 (85,2)	104 (84.6)	63 (86.3)	0,739
M	29 (14,8)	19 (13.4)	10 (13.7)
Number of pills taken for self-poisoning, median, (25-75 p)	14 (10-30)	20 (10-36)	12(8-16)	<0.001
> 1 type drug, n (%)	97 (49.5)	63 (51.2)	34 (46.6)	0.530
Self-harm scarring (+), n (%)	189 (96,4)	117 (95,1)	72 (98,6)	0,261
First suicide attempt (+), n (%)	175 (89,3)	109 (88.6)	66 (90.4)	0,695
Glasgow Coma Score <15, n (%)	6 (3.1)	4 (3.3)	2(2.7)	>0.999
Pathological findings on physical examination, n (%)	10 (5.1)	6 (4.9)	4 (5.5)	>0.999
Cardiacarrhythmia on ECG, n (%)	5 (2.6)	2 (1.6)	3 (4.1)	0.363
Abnormality in any laboratory result on admission, n (%)	44 (22.4)	32 (26)	12 (16.4)	0.120
Pathological findings on first pediatric psychological consultation, n (%)	152 (77.6)	112 (91.1)	40 (54.8)	<0.001

## DISCUSSION

In this retrospective analysis, we found that most SAs in the study population were performed by female children and adolescents. Antidepressants were most frequently chosen. Approximately half of the patients took multiple drugs. The number of pills taken and the incidence of pathological findings at the first paediatric psychiatric consultation were higher in hospitalised than in non-hospitalised patients. We found no significant difference between the patients discharged from the PED and the patients hospitalised in terms of clinical or laboratory parameters.

Worldwide, approximately 200,000 adolescents lose their lives due to suicide every year,^3^ making suicide one of the most important causes of morbidity and mortality in this age group.³ SA is one of the most important problems in PEDs.^8^

In the literature on paediatric patients who attempt suicide, the ages considered range from 13 to 18 years.^9^-^11^ Most studies have reported higher numbers of female than male patients.^3^,^9^,^10^ However the proportion of females in our study (approximately 85%) was much higher than that reported in the literature. The reason for this difference may be that we included only children who attempted suicide by taking an overdose of pills.

Some authors have reported that the rate of previous SA was high among children admitted to the emergency department.^3^,^10^,^12^ However, these results do not correspond with our data. In our study, the rate of previous SA was only 10%. This difference may arise from the fact that our study covered only one year. Additionally, most of the published studies were carried out in developed countries. Our country is a developing nation, and this may affect our findings.

Antidepressants, non-steroidal anti-inflammatory drugs, and analgesics are mainly chosen in self-poisoning attempts.^13^-^15^ Our results were similar to the literature in this regard. Additionally, flu medications were used by one our of every four patients in our study group. We found no significant difference in the hospitalization rate, based on the type of drug used in SAs.

Various drugs lead to different clinical results, and the most severe clinical outcomes are dose-related.^16^-^18^ The need for hospitalization varies according to the clinical situation and risk factors.^9^,^10^,^19^ Psychiatric problems are common in patients being treated for non-fatal SAs.^20^,^21^,^22^ In our study, the physical examination and laboratory tests revealed no abnormalities in most patients. However, abnormal psychiatric findings were seen in the majority of children in the acute phase, with major depression being the most common disorder. The number of pills used in SAs was much higher, and abnormal psychiatric findings in the acute phase were much more common in hospitalized patients compared with discharged patients, among children who performed non-fatal SA using pills. The abnormal clinical and laboratory findings did not differ between the groups. These results indicated that the amount of drugs consumed, as determined from the medical history, and the results of the paediatric psychiatry consultation during the acute phase of poisoning may be important parameters affecting the decision by PED physicians regarding whether to hospitalize. These findings were likely related to two important considerations. One of these was the features of our study group, which consisted of children with non-fatal, moderate to severe poisoning. Secondly, our PED is a training center; thus, most patients with moderate to severe clinical stages received appropriate medical care in our emergency department, as they would in other top-tier emergency departments around the world^19^; furthermore, we were able to follow them in the observation unit without hospitalization for the first 24 h. Hence, there was usually no need to hospitalize patients based on abnormal clinical findings at admission. The remaining factors affecting the hospitalization decision in the case of children who performed non-fatal SA via pills were the risks posed by a severe clinical situation (the number of pills used in SA) and the risk of SA recurrence (presence of psychiatric problems during the acute phase of poisoning).

### Limitations of the study

First, the retrospective character of the study is an important limitation. Second, our results were from only one center; however, the number of patients included in the study was substantial. Finally, prospective multi-center studies will better illuminate the factors that affect the hospitalization decision in PED.

In conclusion, in a top-tier PED, the factors affecting the disposition decision for children who performed non-fatal SA via pill overdose were the amount of medication used for SA and psychiatric disorders as determined by a paediatric psychiatrist during the acute phase.
